# Supplementary material for: Are carbon emissions trading and green financial instruments synergistic? -Comprehensive quantitative research based on content analysis
Source: PLoS One. 2024 Mar 7;19(3):e0298601. doi: 10.1371/journal.pone.0298601 (PMC10919601; doi:10.1371/journal.pone.0298601)
Supplement: S1 Data — (ZIP) [file pone.0298601.s001.zip › Data/地级市Z_F工作报告.zip!/─┌├╔╣┼/░═╤σ─╫╢√╩╨/└┤╫╘╣1⁄2╓┌║┼í╛┬φ┐╦╩2╛▌═°í┐_2019.docx]

各位代表：

现在，我代表市人民政府向大会报告工作，请予审议，并请市政协委员和列席会议的同志提出意见。

一、2018年工作回顾

2018年是贯彻党的十九大精神的开局之年，也是本届政府履职的第一年。我们坚持以习近平新时代中国特色社会主义思想为指导，全面落实党中央国务院、自治区党委政府各项决策部署，认真贯彻市委四届四次全会和全市重点工作推进会精神，坚持稳中求进工作总基调，克难攻坚、真抓实干，经济社会发展态势总体平稳、稳中向好。预计全市地区生产总值完成790亿元，增长5.5%；一般公共预算收入完成55.5亿元，剔除一次性因素增长6.3%；城乡居民人均可支配收入分别完成30400元、16900元，增长7.5%、8%。

一年来，我们主要做了以下工作：

（一）坚持转方式、调结构，新旧动能转换步伐加快。紧扣市委“五区共建”、建成现代化生态田园城市的发展定位和塞上江南、绿色崛起的奋斗目标，高水平编制了《巴彦淖尔高质量发展规划》，为全市长远发展提供了科学遵循。举办了中国新型城镇化（巴彦淖尔）现代化生态田园城市高层论坛。全市“十三五”规划纲要中期评估和调整基本完成。全力建设河套全域绿色有机高端农畜产品生产加工输出基地，成功注册“天赋河套”区域公用品牌，荣登2018中国区域农业形象品牌影响力排行榜亚军，首批授权的6家企业产销两旺，产品受到消费者的青睐和好评。与中国标准化研究院合作，建设标准化实验室；市食品药品检验所通过国家实验室能力认可，出具数据实现国际互认。制定完善农畜产品生产技术标准185项，市级食品质量安全智慧追溯平台建成投用，130家企业纳入自治区农畜产品质量安全可追溯体系。新建了一批田园综合体，美国金伯利农庄落地建设，国家农高区创建工作有序推进。工业经济提质增效，预计规模以上工业企业主营业务收入增长6.8%，企业利润增长15.8%。全年减轻企业税费负担22.8亿元、用电成本4.5亿元。恒嘉蓝宝石一期、东立多晶硅一期、易中易冷链物流一期、旭一牧业和金草原肉牛加工、康斯特果胶等项目建成投产，中核龙腾光热、黑猫煤化工、盾安多晶硅三期、中粮面粉加工等项目加快推进。新增多晶硅产能6000吨；新增风光电装机101万千瓦，累计达到487万千瓦；新兴产业占规模以上工业比重达到16.8%。现代服务业发展势头强劲，对经济增长的贡献率达到70%以上。成功举办世界向日葵产业发展论坛暨花季旅游文化节、第二届内蒙古绿色农畜产品（巴彦淖尔）国际博览会、国际马拉松赛、国际骆驼文化旅游节、乌梁素海国际冰雪旅游节、中蒙俄国际越野拉力赛等活动，全年接待游客664万人次，实现旅游收入68.4亿元，分别增长20.3%、21.8%。开通兰州、重庆2条定期航线和哈尔滨、博鳌2条旅游航线，旅客吞吐量增长25%。电商产业园和50户以上村组电商服务网点实现旗县区全覆盖，全区首家电子商务大数据中心启动运营。金融机构存贷款余额分别达到1050亿元和890亿元。

（二）坚持修生态、强基础，城乡环境明显改善。认真贯彻习近平总书记关于“一湖两海”治理的重要指示精神，坚持系统思维、多点发力，山水林田湖草沙综合治理取得实质性进展。规划投资57.46亿元的乌梁素海流域山水林田湖草生态保护修复试点工程，通过竞争性评审，以第一名成绩在20个省区市中脱颖而出，获得国家项目支持，10亿元基础奖补资金已拨付到位。“四控”行动破题起步，点源污水“零入海”工程加快推进，充分利用黄河凌汛水和山洪水，实现生态补水5.94亿立方米，乌梁素海水质持续改善。河（湖、渠）长制全面推行，落实各级河（湖、渠）长1193名。完成盐碱地改良83万亩，成功举办全国盐碱地改良技术高峰论坛，五原县5万亩改盐增草（饲）兴牧产业园成为全国样板区。不等不靠，自加压力，大力推进乌拉山矿山治理修复工程，完成治理面积20.6平方公里。乌兰布和沙漠治理加快推进，成为全国防沙治沙综合示范区。发放草原生态补奖资金3.25亿元，完成林业生态建设56万亩，成功承办全区推进国土绿化现场会。我市绿色可持续发展模式入选可持续金融国际会议示范案例。加快修编乡村振兴、“城市双修”、双河区和甘其毛都口岸规划。完成14处历史建筑确定和公布工作。新改建城镇道路48公里，改造老旧小区138万平方米、平房区7427套，开工棚户区改造12877套，临河区北边渠拆迁完成2102户。双河区基础设施建设稳步推进。全市新增园林绿地面积3274亩。农村牧区人居环境三年整治行动全面推开，建成农村集镇污水处理厂10个，完成村庄环境综合整治100个，改造厕所1.44万户，3247个村庄建立健全了后续管护机制。灌区续建配套与节水改造、黄河二期防洪等工程扎实推进。国道242甘其毛都至临河一级公路、京新高速陕坝连接线、省道311建丰农场至建设段建成通车，新改建农村牧区公路1500公里，五原县成为全国“四好农村路”示范县。

（三）坚持抓重点、补短板，三大攻坚战扎实推进。把打好三大攻坚战作为重大政治任务，举全市之力抓紧抓实，取得阶段性成果。全年化解政府债务108.1亿元，完成年度目标任务的138.1%，“十个全覆盖”10项工程拖欠工程款23.23亿元全部化解。严格规范投融资行为，清理整顿违法违规金融行为，金融风险平稳可控。乌拉特中旗、杭锦后旗、五原县、磴口县脱贫摘帽，乌拉特前旗接受了自治区脱贫退出评估验收，3316名贫困人口实现脱贫，贫困发生率下降到0.04%。中央环保督察及“回头看”反馈问题整改工作按期有序推进，一批群众反映强烈的环境问题得到有效整治。蓝天、碧水、净土行动全面开展，中心城区空气质量优良率达到86.7%，国控断面水质稳定达标，环境质量持续改善。

（四）坚持抓改革、促开放，发展动力活力不断增强。以庆祝改革开放40周年为契机，持续深化改革和扩大开放。习近平总书记嘱托的三项重点改革向纵深推进，供给侧结构性改革不断深化。“放管服”改革走在全区前列，营商环境评价全区排名第一。“互联网+政务服务”网上办事大厅启动运行，70%的政务服务事项实现在线办理；337项公共服务事项进驻市政务服务中心，实现了应进全进。企业投资项目和工业项目审批时限由过去200多天减少为112天和110天，提前实现“减半”目标。农村土地确权、国有林场改革基本完成，农村集体产权制度改革被列为全国试点，黄河干流水权置换试点工程通过国家核验。村民小组“微治理”、五人小组工作法等改革创新了农村牧区基层治理模式。水务管理体制、国企管理体制改革积极推进，教育医疗、财政金融、生态文明等改革稳步实施。全市对外贸易进出口总额突破250亿元，跃居全区首位。以口岸开发开放为重点、提高对外开放水平的做法，受到国务院通报表扬。对蒙合作更加紧密，成功接待了蒙古国总理、矿业部长等高层访问，与蒙方签署了1000万吨主焦煤合作协议。口岸智能化通关系统投入使用，“七进七出”矿能通道加快建设，口岸过货量突破1900万吨，完成海关税收35亿元，再创历史新高。甘其毛都至嘎顺苏海图国际道路客运班线启动运营，开通了全区首列中欧农产品班列，唐山港巴彦淖尔内陆港开通运营。举办了首届内蒙古西部国际商贸展洽会。深入开展“招商引资年”活动，签署了乌梁素海流域生态修复绿色产业开发基金、中国烟草基金、绿色金融等一批合作协议，全年引进国内到位资金207亿元。

（五）坚持惠民生、增福祉，群众获得感不断增强。牢固树立以人民为中心的发展思想，各级财政投入民生支出201亿元，占一般公共预算支出的75.4%。新增城镇就业1.1万人，农牧民转移就业13.3万人次，城镇登记失业率控制在3.87%以内。为全市60周岁以上老年人购买意外伤害保险。依法维护农牧民工合法权益，欠薪案件实现动态清零。新增国家高新技术企业22家，新建院士专家工作站4个。引进高层次急需紧缺人才185人，驻京人才工作站挂牌成立。组建医联体18个，在全区率先建成了市域“半小时急救圈”空地一体化救援体系。新改扩建中小学、幼儿园27个，河套学院新校区、内蒙古美术职业学院新校区建成投用，市委党校新校区主体完工。全面推行“明厨亮灶”工程，新改造提升餐饮企业3860家。承办了首届全区乌兰牧骑新人新作大赛，举办了全市乌兰牧骑艺术节。大型革命历史电视剧《鸿雁》、农村励志题材电视剧《鸿雁故乡》、反映河套人民早期创业纪录片《百年流淌杨家河》拍摄完成；侯卫东官场笔记《片警宝音》入选庆祝改革开放40周年9部重点影片，入围第14届中美电影节“金天使奖”。五原抗战纪念园建成开馆。新改建“儿童之家”20所、“一旗一馆”4个，全民健身中心实现旗县区全覆盖。城乡公交改革取得新进展。面对历史罕见的旱灾和洪涝灾害，全市上下团结一心、众志成城，取得抢险救灾斗争的全面胜利。扫黑除恶专项斗争取得阶段性战果，社会治安形势稳定，“爱民固边直通车”入选2018年自治区10大法治事件。非洲猪瘟疫情“零发生”。食品药品安全、信访维稳、安全生产形势总体平稳，民族团结、社会和谐、边疆安宁的良好局面进一步巩固。

（六）坚持转作风、提效能，政府自身建设进一步加强。深入学习贯彻习近平新时代中国特色社会主义思想和党的十九大精神，自觉树牢“四个意识”，坚定“四个自信”，坚决做到“两个维护”，始终在思想上政治上行动上同以习近平同志为核心的党中央保持高度一致。认真落实全面从严治党责任，严格落实中央八项规定及实施细则，驰而不息纠正“四风”，各级政府工作人员主动作为、担当实干的劲头明显增强。自觉接受人大依法监督和政协民主监督，建议和提案办复率均为100%。坚持依法行政，严格执行重大决策法定程序，组建了政府法律顾问团和专家智库。积极开展地方立法工作，《巴彦淖尔市城市市容和环境卫生管理条例》颁布。扎实开展“七五”普法，全社会法治意识不断提升。全力支持纪委监委工作，加大审计监督工作力度，政府系统廉政建设深入推进，治理能力和水平不断提升。

各位代表，事非经过不知难。过去一年的成绩来之不易，是以习近平同志为核心的党中央坚强领导的结果，是习近平新时代中国特色社会主义思想引航定向、伟大实践的结果，是自治区党委、政府和市委正确领导的结果，是市人大、政协和社会各界监督支持的结果，是全市各族人民守望相助、团结奋斗的结果。在此，我代表市人民政府，向全市各族人民，向各位人大代表、政协委员和社会各界人士，表示衷心的感谢！向所有为巴彦淖尔建设和发展作出贡献的同志们、朋友们，表示崇高的敬意！

各位代表，成绩是前进的基石，问题是努力的方向。我们清醒地认识到，经济社会发展和政府工作还存在一些突出问题。主要有：一是三大攻坚战还有较多薄弱环节。我市财力规模小，收支矛盾突出，可用的化债资金资源匮乏，化债面临较大压力。产业扶贫相对滞后，巩固脱贫成果还需持续用力。节能降耗形势严峻，乌梁素海流域生态环境修复治理任重道远，中央环保督察及“回头看”反馈问题整改工作还需加大落实力度。二是产业发展不充分，重大项目储备不足，新兴产业规模还比较小，科技创新能力亟待提高，营商环境有待进一步优化。三是与群众对美好生活的需求相比，在教育、医疗、养老、文化、体育、物业服务等民生领域还存在一定短板，城乡基础设施和公共服务还不够完善，城市精细化管理和服务水平有待进一步提高。四是政府自身建设还需进一步加强，一些干部思想不够解放、视野不够开阔、创新意识不够强，缺乏担当作为精神，能力素质有待进一步提升。对于这些问题，我们必须清醒认识，切实加以解决。

二、2019年重点工作

2019年是新中国成立70周年，也是全面建成小康社会关键之年。刚刚闭幕的市委四届六次全会暨全市经济工作会议，认真贯彻中央经济工作会议和自治区党委十届八次全会暨全区经济工作会议精神，紧紧围绕“五区共建”、建成现代化生态田园城市的发展定位和塞上江南、绿色崛起的奋斗目标，保持战略定力，加强顶层设计，运用战略思维、创新思维、系统思维，提出了今年全市工作的总体要求和重点任务，为我们做好各项工作提供了重要遵循。我们一定要提高政治站位，全面履职尽责，奋力开创高质量发展的新局面。

今年政府工作的总体要求是：以习近平新时代中国特色社会主义思想为指导，认真贯彻党的十九大、十九届二中、三中全会、中央经济工作会议、自治区党委十届八次全会和市委四届六次全会精神，坚持稳中求进工作总基调，坚持新发展理念，坚持推动高质量发展，坚持以供给侧结构性改革为主线，坚持改革开放，坚定不移走生态治理、产业发展和民生改善相结合的路子，全力以赴推动绿色产业集聚区、生态文明示范区、乡村振兴样板区、对蒙开放先行区、美好生活共享区建设，为全面建成小康社会、建设现代化生态田园城市打下坚实基础，以优异成绩庆祝新中国成立70周年。

今年主要预期目标总体上以稳为主，突出绿色高质量发展导向。地区生产总值增长5.5%，城乡居民人均可支配收入分别增长7.5%、8.5%，城镇登记失业率控制在4%以内，单位地区生产总值能耗完成自治区下达的目标。

实现上述目标，我们要重点抓好以下工作。

（一）坚决打好三大攻坚战。三大攻坚战，越往后难度越大。我们要切实提高政治站位，聚焦重点难点，全面精准发力，确保完成年度目标任务。

积极主动防范化解重大风险。全面加强政府债务管理，打好预算安排、资产重组、资源盘活等“组合拳”，更多采用市场化办法化债，确保完成年度化债任务。依法规范政府与社会资本合作、政府投资基金、政府购买公共服务等行为，坚决杜绝违法违规举债。加快政府性投融资平台市场化转型，提高实体化经营发展能力。建立金融信息化监管服务系统，规范金融市场管理，打击非法集资、金融诈骗、农村牧区高利贷等违法违规金融活动，持续优化金融环境。

以绣花功夫抓好脱贫攻坚。深入开展脱贫攻坚三年行动，夯实责任，强化精准帮扶，确保新识别和返贫人口全部脱贫，乌拉特前旗脱贫摘帽。已摘帽旗县保持攻坚态势，巩固提升脱贫成效，确保脱贫攻坚继续走在全区前列。积极整合各类涉农涉牧资金，加大金融扶贫力度，全市扶贫投入不降低。把脱贫攻坚与乡村振兴战略有机衔接起来，把产业扶贫作为稳定脱贫的治本之策，建设一批产业扶贫园区，大力推广入股分红、资产租赁、利息分红等资产收益模式，发展壮大村集体经济，让贫困群众获得更多稳定收益。按照中央和自治区部署，统筹整合各方力量，研究解决好收入水平略高于建档立卡贫困户的群众缺乏政策支持的问题。

标本兼治推进污染防治。扎实抓好中央环保督察及“回头看”反馈问题整改工作，按时限完成整改销号。打好蓝天保卫战，全面完成电力行业超低排放改造，淘汰燃煤小锅炉，探索推广分布式光伏取暖，着力解决工业排放、粉尘污染等问题。深入实施水污染防治行动，完成饮用水水源地保护区综合治理，完成326个地下油灌防渗改造，保障人民群众饮用水安全。全面推进垃圾分类管理和处置，规范餐厨垃圾处理。实施临河区、乌拉特前旗畜禽粪污资源化利用整县推进项目。完成第二次全国污染源普查工作。持续开展环境治理专项整治行动，切实解决油烟污染、露天烧烤、噪声扰民等群众反映强烈的环境问题。妥善处理好环境执法与企业发展、群众生活的关系。

（二）统筹推进山水林田湖草综合治理。这是习近平总书记交给我们的重大政治任务，也是我市的重大发展战略，事关长远、事关全局，必须举全市之力推动落实。

全面实施乌梁素海流域山水林田湖草生态保护修复试点工程。认真落实乌梁素海综合治理“1个意见”和“13个配套办法”，压实责任，强力推进，以更高的标准、更严的要求，全力抓好贯彻落实。用好用足国家和自治区资金，整合各类生态保护专项和相关资金，积极吸引社会资本投入，设立乌梁素海流域生态治理与绿色产业发展基金，确保完成年度建设任务。科学制定7大类工程、38个子项目实施计划，明确时间表、路线图、责任人，引入国际现代工程管理模式，高标准实施、高效率推进、全过程监管，打造看得见、摸得着、可复制的山水林田湖草沙生态系统保护修复样板。

积极抓好重点生态区域综合治理。加大乌梁素海生态治理力度，市财政安排奖励资金1亿元，每个旗县区配套不少于1000万元，用于补贴农业面源污染治理，年内化肥、农药利用率分别提高2.6和2个百分点；开展废旧地膜“以旧换新”和农药包装物回收行动，全面推广使用国标地膜。推进城镇、工业园区及散排企业废水循环利用，中水回用率达到100%。实施乌梁素海湖区增容、水道疏浚、湿地修复等项目，完成生态补水3.5亿立方米以上。全面落实河（湖、渠）长制，有名有实地推进工作，开展“清四乱”专项行动，建设清洁型、生态型河湖。加强地下水超采区治理。实施改盐增草（饲）兴牧工程50万亩，高标准完成乌梁素海周边5万亩试验示范项目。按照绿色矿山建设标准，持续深入抓好乌拉山及周边地质环境综合整治，完成治理面积6平方公里。实施天然林保护、京津风沙源治理等重点工程，完成林业生态建设78万亩。落实草原生态补奖政策，加强草原生态修复和保护。编制完成《巴彦淖尔市防沙治沙综合示范区规划》，积极推进沙产业园、沙生植物园和光伏特色小镇“三位一体”建设。

（三）全力推进河套全域绿色有机高端农畜产品生产加工输出基地建设。产业兴旺是乡村振兴的重要基础。我们要坚持质量兴农、绿色兴农、品牌强农、科技强农，以强有力的举措，推动农牧业绿色高质量发展。

打响“天赋河套”公用品牌。出台“天赋河套”品牌授权准入、监督管理、奖励惩处、授权退出、品牌推介招商奖补等办法，进一步健全质量标准和可追溯体系，对符合条件的产品优先授权使用“天赋河套”品牌。全面启动“天赋河套”品牌运营总部基地建设。在区域中心城市设立“天赋河套”产品精品馆、体验店、直销店。利用各类媒体、展销会、文体旅游活动，全方位推介“天赋河套”品牌，提高品牌的知名度和影响力，让“天赋河套”品牌走向全国、走向世界。建设“天赋河套”标准化研究院，制定全市主要和特色农畜产品标准。加大品牌保护力度，严厉打击假冒伪劣，让“天赋河套”品牌越擦越亮。继续办好第三届内蒙古绿色农畜产品（巴彦淖尔）国际博览会。

调整优化农牧业结构。稳定粮食生产能力，扩大优质小麦种植面积，青贮玉米面积达到80万亩，新增设施农业1.5万亩，扩大优质牧草、中药材种植面积，加大新品种、新技术推广应用力度。坚持稳羊增牛发展方向，适度发展骆驼、肉驴、水产等特色养殖，振兴奶业、羊绒等产业，建设国家级区域性肉羊良种繁育基地。有序扩大经济林种植面积，科学发展林果业，新增面积2万亩以上。

推进农村牧区产业融合发展。编制完成《巴彦淖尔市农牧业绿色发展规划》，细化编制粮油、肉乳绒、果蔬、蒙中药材、饲草、生物质能等优势特色产业实施方案，引进和培育壮大龙头企业，推进全产业链发展、一体化经营。积极培育产业化联合体，建设一批标准化现代园区和生态牧场。全力做好国家农高区申报创建工作，推动金伯利农庄一期建成运营，启动二期建设项目，发挥示范带动作用。建设彤锣湾、鲜农、塔尔湖、三道桥、同和太等一批重点田园综合体和草原牧业综合体。开拓农畜产品销售市场，建设深港澳绿色有机果蔬供应基地。抓好国家有机农产品认证示范县创建工作。加大“三品一标”产品认证力度。推进农牧业与电商、文旅等产业深度融合。强化科技示范园区辐射带动作用，每个旗县区至少集中打造1个万亩以上高标准示范园。创新土地流转模式，发展适度规模经营。大力培育农牧民合作社、家庭农牧场等新型经营主体，引导小农户、小生产更好地对接大市场。依托现有各类园区建设农业创新创业孵化基地，落实金融服务、税费减免、创业培训等优惠政策，引导和支持大学生、退伍军人、科技人员返乡创业，强化乡村振兴的人才支撑。

（四）加快工业经济转型升级步伐。坚持盘活存量和做大总量并重、改造升级“老字号”和培育壮大“新字号”齐抓，促进工业经济提质增效。

推动传统产业优化升级。运用高新技术和先进适用技术改造农畜产品加工、矿山冶金、能源化工等产业，不断增强产业竞争力。全力推进黑猫煤化工一期、神华一期二线等项目建设，扩大进口煤铜资源落地加工能力；加快实施中粮面粉加工、润海源肉鸡加工、蒙驼骆驼产业、易中易冷链物流二期等项目，实现全产业链发展，提高产业附加值；积极推动国城硫铁钛资源综合利用、蒙润硫酸钾肥、力景华荣有机肥二期等项目建设，提高资源循环利用水平；加快华拓矿业等企业节能技改步伐，促进企业节本增效，提升产业层次和水平。

大力发展战略性新兴产业。推动我市千万千瓦现代能源基地项目优先纳入自治区现代能源经济发展战略规划。积极争取光伏“领跑者”基地、第二批光热示范项目。启动上海庙至山东特高压外送通道配套160万千瓦风电项目，推动中核龙腾10万千瓦光热、四华20万千瓦风电、神华国能20万千瓦风电、杭龙生物质热电联产等项目建成投产，争取五原县5万千瓦光伏智慧新农村平价上网项目列入自治区试验示范项目范围。积极推动微电网试点建设。实施盾安多晶硅三期、东立多晶硅二期、恒嘉蓝宝石二期、科晟电子单晶硅等项目，发展壮大新材料产业。编制石墨产业发展规划，规划建设高科技石墨特色小镇。大力发展数字经济，加快编制数字巴彦淖尔专项规划，积极搭建乌梁素海流域生态环境大数据平台，在乌拉特后旗规划建设数字经济创新试验区，积极推动中科院与我市开展大数据及物联网产业发展战略合作。围绕生态治理和资源综合循环利用，谋划实施一批生态产业项目，推动生态产业化、产业生态化。

强化工业经济运行调度。统筹用好电力扶持、节能技改、税收优惠等政策措施，帮助企业解决原料供应、项目融资、产品销售等实际问题，促进企业满负荷生产。加强节能监测预警，严格控制能耗过快增长。推进授权使用“天赋河套”品牌的农畜产品加工企业打捆进入电力多边交易市场，享受优惠电价。实施园区振兴战略，积极争取国家和自治区专项扶持资金，完善园区基础设施，提高园区承载能力。

加大招商引资力度。围绕全市高质量发展规划，严格遵照全市产业布局要求，更新完善招商引资项目库。创新招商引资方式，采取项目招商、园区招商、金融招商、以商招商、产业链招商、委托招商等多种形式，充分发挥各地商会作用，拓宽招商引资渠道，增强招商引资工作的精准度和实效性。狠抓项目跟踪对接，提高项目履约率和资金到位率。

（五）大力发展现代服务业。服务业是我市培育新动能的巨大潜力所在。我们要推动文化旅游、现代金融、电商物流等产业提档加速，增强服务业对经济发展的支撑作用。

推动文体旅产业融合发展。充分挖掘地区文化内涵，以文强旅、以旅兴文，形成叠加和倍增效应。加快磴口黄河风情特色小镇、五原葵花主题公园等旅游景区建设，力争乌兰布和沙漠旅游区成为国家4A级景区。围绕双河区总体规划，加快黄河国家湿地公园建设。依托国道242甘其毛都至临河一级公路规划建设精品旅游线路和自驾车营地。大力发展乡村旅游，集中打造一批特色鲜明的乡村旅游示范点。继续办好国际马拉松赛、中蒙俄国际越野拉力赛、黄河湿地龙舟赛、沿黄河公路自行车赛、黄河铁人三项赛、乌梁素海国际冰雪旅游节、国际骆驼文化旅游节、向日葵花季旅游文化节等活动，增强旅游文化的品牌效应，力争全年接待游客人数和旅游收入进一步提升。

发挥金融助力作用。大力发展普惠金融，鼓励金融机构创新信贷产品，探索开展动产抵押贷款、农畜产品价格指数、信用保证保险等业务，鼓励企业通过供应链金融、无形资产抵质押、私募股权投资、股权众筹等模式融资，力争信贷总量达到1000亿元以上，社会融资规模增量达到100亿元以上。积极推动多层次资本市场融资，推进5家企业在新三板挂牌。加快中国烟草基金项目前期工作进度。推动甘其毛都口岸和河套水务公司收益权资产证券化项目落地。稳妥推进乌拉特中旗、乌拉特后旗农村信用社改制工作。力争举办国际可持续金融会议及全球高校可持续投资实践竞赛，积极争取德国复兴信贷银行、法国开发署等国际贷款，全力推进首笔绿色债券发行。

提升电商物流产业层次。以“天赋河套”品牌推广为主线，加强与京东、阿里巴巴等电商平台合作，培育壮大本土电商品牌，积极发展跨境电商，力争电商交易额增长30%以上，创建国家电子商务示范城市。推进天津渤海商品交易所合作共建特色商品交易中心，推动农畜产品线上线下融合发展。深化全国网络市场监管与服务示范区创建工作，维护网络市场秩序和消费者合法权益。加强农牧民电商技能培训，开展“电商村”建设。大力促进甩挂运输、冷链运输、多式联运等物流业态发展，开工建设临河铁路物流基地，抓好B型保税物流中心发展运营。

发展壮大民营经济。认真贯彻习近平总书记在民营企业座谈会上的重要讲话精神，不折不扣落实自治区促进民营经济高质量发展的26条措施，结合我市实际制定出台具体的实施办法。帮助困难企业积极争取自治区民营企业流动性风险防控基金和纾困发展基金支持，市本级安排支持民营经济发展专项资金，尽最大努力帮助企业应急解难。推动银行业金融机构支持民营企业发展，对出现困难的民营企业，实施差异化信贷政策。积极争取自治区融资担保基金支持，整合壮大市级融资担保机构实力。依法规范民营企业融资行为。全面推行市场准入负面清单，严格规范涉企执法检查。大力弘扬企业家精神，构建亲清新型政商关系，依法保护企业家合法权益，让企业家在巴彦淖尔放心投资、舒心创业、安心发展。各级政府要恪守诚信，依法依规作出的承诺要严格兑现。

（六）深化改革扩大开放。改革开放只有进行时，没有完成时。我们要以深化改革破解发展瓶颈，以扩大开放拓展发展空间，激发经济发展的动力和活力。

加快重点领域改革。扎实推进习近平总书记嘱托的三项重点改革，推广股份合作、服务协作等紧密型农企利益联结机制。全面落实“三线一单”管理制度。口岸“单一窗口”应用率要达到100%。深化农村牧区综合改革，稳妥推进“三权分置”改革。加快推进集体产权制度改革试点，开展身份确认、股权量化等工作，10月底前完成改革任务。深化水务体制改革，推进政事企分开，激发水务系统内部活力，促进水费公平负担。实施财政体制改革，合理划分市与旗县区财政事权和支出责任。按照自治区党委和市委的部署，完成政府机构改革。深化公立医院等领域改革，分类推进事业单位改革，推动试点改革和自主探索改革。

以更大决心、更大力度推进“放管服”改革。深化政务服务“一网、一门、一次”改革，深入推进“互联网+政务服务”工程，筹建政务服务热线平台和四级互动平台，年内85%以上的事项实现网上办理。企业投资项目审批由政务服务中心一窗受理，探索开展多评合一、多规合一、并联审图、联合踏勘、联合验收，推行职能部门代跑代办制度，进一步提升审批服务效能、方便企业群众办事。全面推行“证照分离”改革，让更多市场主体持照即可经营。学习借鉴先进地区经验，在工业园区探索开展“标准地+承诺制”试点工作。加强四级政务服务体系建设，在苏木乡镇、街道社区设置自助服务终端，加强基层便民服务中心（代办点）规范化建设，让群众少跑路。持续深化综合行政执法改革。加强信用体系建设，健全失信联合惩戒机制，让守信者处处受益、失信者寸步难行。

提高对外开放水平。以中蒙建交70周年和甘其毛都口岸实现常年开放10周年为契机，加快推进中蒙（甘其毛都—嘎顺苏海图）国际产能合作区、国家级重点开发开放试验区等合作平台建设，推动境内外资源合作开发。加快甘其毛都口岸机构设置和职能完善，提高政务服务水平。实施“口岸基础设施建设提升年”行动，加快“七进七出”矿能通道、联检大楼改扩建、驰恒煤炭环保型监管库等项目建设，提升口岸通关能力，力争口岸货运量达到2500万吨以上。发展口岸金融，建立多元化的国际贸易结算渠道和结算方式。推动边民互市贸易区建设，促进口岸贸易多元化发展。加快实施口岸供水及管网配套工程。加强口岸医院建设，提升医疗服务水平。办好第二届内蒙古西部国际商贸展洽会，推动中欧农产品班列常态化运营。深化区域经济合作，主动对接、积极配合做好沿黄生态经济带建设规划编制工作。

（七）统筹推进城乡协调发展。紧紧围绕建设现代化生态田园城市的目标，不断改善城乡人居环境，加大基础设施建设投入力度，提升协调发展水平。

提升城镇功能品质。加强城镇基础设施建设和旧城区改造，加大城镇道路、地下管网、背街巷道、老旧小区改造建设力度，全市新增集中供热面积70万平方米，燃气普及率达到90%以上。用好用足棚改专项债券资金，因地制宜，加大棚户区改造力度。坚持宜拆则拆、宜改则改，在具备条件的平房区，推行水、电、气、暖、厕综合配套改造。通过市场化方式，吸引有实力的企业，全面实施临河区北边渠水源路至建设路段改造。采取有力措施，加快解决群众普遍关心的房产证办理、回迁安置等问题，大力整治房地产开发遗留的“夹心房”问题。建立房地产开发企业“黑名单”制度，名单内企业一律不得新上房地产项目。加大城镇园林绿化建设力度。积极推进双河区建设，争取土储、棚改等专项债券，规划推进黄河全流域文化博物馆、乌拉特文化创意产业园、世界岩刻博物馆、河套特色农畜产品展馆等文旅产业项目，配套推进河套餐饮文化、乌拉特民族手工艺街区。认真落实《巴彦淖尔市城市市容和环境卫生管理条例》，全面推行“街长制”管理模式，提升城市精细化管理水平，依法治理占道经营、掏门改窗、私搭乱建等问题，打造美丽街道、靓丽街区。优化公交线网布局，改善群众出行条件。规范物业管理，提升服务水平。

加快美丽乡村建设。学习借鉴浙江“千村示范、万村整治”工程经验，扎实开展农村牧区人居环境整治三年行动，全力打好乡村振兴第一场硬仗，新建14个镇村污水处理厂、21个垃圾转运站，确保1.2万户农牧民使用上清洁的水冲厕所。完成G7高速和国道242甘其毛都至临河一级公路绿化，实施村庄绿化100个。开展“大棚房”问题专项整治行动，从严整治农地非农化问题。建设“四好”农村牧区公路600公里，坚持综合施策，标本兼治，坚决整治超载超限。实施农村牧区安全饮水工程，改善4万户、12万人饮水质量，彻底解决边境一线牧区人畜饮水安全和“地方病”问题。广泛开展文明镇村、星级文明户、最美家庭、道德模范等评选活动，推动移风易俗，树立文明乡风。

加强重大基础设施建设。扎实做好包银高铁前期工作，加快国道331乌珠尔至北银根三级公路、国道335乌不浪口至乌根高勒二级公路、国道335呼和温都尔至巴彦努如二级公路、省道311朝阳至建丰农场二级公路等项目建设，推进省道311建设至查素沟口二级公路建成通车，争取省道215乌不浪口至新安段开工建设。争取甘其毛都口岸经海流图至乌拉山公路列入自治区地方高速公路网规划，先期实施甘其毛都口岸经海流图段项目。推进省道213团结渠至临河公路建设。加快建设灌区续建配套与节水改造、水库除险加固、水土保持、防汛抗旱、中小河流治理等重点项目。实施河湖水系连通和沙漠治理引水工程，充分利用黄河凌汛水资源补给境内湖泊，变水患为水利。加强电力基础设施建设，实施新安、黑柳子、忠义220千伏和晨光、丰州110千伏等43项输变电工程。认真开展“十四五”规划前期研究，有针对性地谋划一批大项目、好项目。

（八）持续增进民生福祉。我们要完善制度、守住底线，既尽力而为、又量力而行，加强普惠性、基础性、兜底性民生建设，让群众有更多获得感、幸福感、安全感。

千方百计促进就业增收。坚持就业优先战略和积极就业政策，促进高校毕业生、城镇困难人员、农牧民工、退役军人等重点群体就业，新增城镇就业9500人，农村牧区转移就业12万人次，确保零就业家庭动态清零。大规模开展职业技能培训，提升劳动者就业创业能力。建设市级标准化创业园3个、示范性创业孵化基地2个。深入实施援企稳岗“护航行动”，充分发挥民营企业、中小微企业就业主渠道作用。实施农牧民增收行动计划，努力增加农牧民经营性、工资性、财产性收入。

提高社会保障水平。稳步提高城乡低保、特困人员供养、孤残儿童、优抚对象补助标准，保障困难群众基本生活。完成机关事业单位养老保险准备期基金清算工作，退休人员养老金社会化发放率达到100%。扩大异地就医结算和单病种结算范围。支持社会力量举办医养结合机构。在牧区建设一批抗旱救灾饲草料储备库。落实农牧民工资支付保障制度，维护农牧民工合法权益。大力发展社会福利和慈善事业，保障妇女、儿童、残疾人合法权益。

深入推进科技创新。加大科技投入力度，支持各类科技创新活动。发挥企业创新主体作用，加强与科研院所、高校合作，完成重大科技成果转化20项以上，新增高新技术企业10家以上、授权专利200件以上。结合优势特色产业发展和国家农高区创建需要，建成一批自治区级以上重点实验室、工程中心、企业研发中心等创新平台载体。建立健全人才引进培养长效机制，引进一批高层次和急需紧缺人才，发挥好专家顾问、科研院所的咨询智囊作用。

全面提升教育质量。认真落实第三期学前教育行动计划，新改扩建幼儿园10所。建成临河区水源路学校、市职业技术培训实训基地和示范性综合实践基地。促进义务教育优质均衡发展，稳妥推进中考改革，深化高中教育教学改革，大力提升教育教学质量。深化民族教育“双语”教学改革，推动优质特色发展。加强教师队伍建设，深化教师准入招聘、编制动态调整、岗位聘用、绩效工资、职称和考核评价制度改革。推进高等教育内涵式发展，做好河套学院本科教学合格评估迎评促建工作。

繁荣发展文化事业。认真落实意识形态工作责任制，大力弘扬社会主义核心价值观。深入实施公共文化服务工程，完善文化活动室、文化大院、电子阅览室、档案馆等基础设施，丰富群众精神文化生活。以新中国成立70周年为主题开展艺术创作活动，推出一批文化精品。办好第二届乌兰牧骑艺术节、“魅力乌拉特”中国西部民歌会。继续做好阴山岩刻申遗工作，加快河套灌区世界灌溉工程遗产申报工作。组织举办“中国岩刻丝路行”——阴山岩刻艺术国际巡展、“天赋河套”——中蒙俄向日葵美术作品名家邀请展等活动，提高河套文化的知名度和影响力。新建临河区市民活动中心等重点群众综合性文化活动场馆。

推进健康巴彦淖尔建设。继续深化医药卫生体制改革，完善“三医”联动机制，落实分级诊疗制度，加快推进现代医院管理制度建设。创新医疗服务模式，重点落实“十项任务”，推动医疗服务机构内涵式发展。增强全民健康意识，倡导健康生活方式，开展全民健身行动，加强全民健康管理。实施健康医疗大数据应用工程，完成70%的城乡居民“电子健康卡”发放应用工作。制定具体措施，推动基层医疗卫生机构面向群众开展基础保健服务。加大骨干全科医生培训力度，40%的基层医疗卫生机构达到国家标准。完成临河区人民医院改造搬迁。扶持蒙中医药事业发展，提高妇幼保健水平。深入开展爱国卫生运动，积极创建国家卫生城市。加强食品药品安全监管，大力推进“明厨亮灶”工程，餐饮企业改造提升率达到90%以上。

提高社会治理水平。学习推广“枫桥经验”，建立健全社会矛盾纠纷多元化解机制，努力把矛盾化解在早、化解在小、化解在基层。推广应用4K智能公共法律服务电视终端，年内实现行政村全覆盖，为农牧民提供优质便捷的公共法律服务。扎实推进平安巴彦淖尔建设，完善立体化社会治安防控体系，升级改造公安视频监控系统。深入开展扫黑除恶专项斗争，持续提升群众安全感。健全防灾减灾救灾体系，提升突发事件应急处置能力和保障水平。加强非洲猪瘟等动物疫病防控，确保不发生区域性重大疫情。严格落实安全生产责任制，坚决防范遏制重特大事故发生。做好退役军人管理服务工作，保障退役军人合法权益。全面贯彻党的民族政策，推进全国民族团结进步示范市创建工作，实施兴边富民行动，大力发展民族文化、民族教育、民族体育事业，促进各民族共同团结奋斗、共同繁荣发展。认真贯彻《宗教事务条例》，依法加强宗教事务管理。

（九）加强政府自身建设。为政之要，首善其身。我们要以政府机构改革为契机，加快职能转变，规范行政行为，优化服务效能，强化责任担当，弘扬总干精神，努力打造人民满意的务实、高效、法治、廉洁政府。

我们要强化政治建设，树牢“四个意识”，坚定“四个自信”，坚决做到“两个维护”，始终在思想上政治上行动上同以习近平同志为核心的党中央保持高度一致。坚持不懈用习近平新时代中国特色社会主义思想武装头脑、指导实践、推动工作，坚决落实党中央国务院、自治区党委政府和市委各项决策部署，确保政令畅通、令行禁止。

我们要强化依法行政，牢固树立宪法法律至上观念，严格按照法定程序和内容履行职责，加快建设法治政府。严格执行重大决策法定程序，积极发挥政府法律顾问作用。严格落实普法责任制和执法责任制。坚持“三重一大”事项向市委常委会报告制度，自觉接受市人大及其常委会的依法监督、市政协的民主监督，做好人大代表建议和政协委员提案办理工作。实现规范性文件提交市人大常委会备案审查全覆盖。积极开展地方立法工作，起草巴彦淖尔市大气、水、土壤污染防治方面的地方性法规草案。

我们要强化实干担当，注重提高执行力，把抓落实作为检验工作成效的重要标准，少说不能为、多想怎么办，用担担子、扣扣子、钉钉子的精神，把定下的事干好，把承诺的事办成。注重培养专业能力，提高专业本领，做到业务熟悉精通，情况了然于胸，做到“政策通”“一口清”，不断提高干部推动高质量发展的能力和水平。以严谨认真的态度，做好第四次全国经济普查工作，进一步把全市的“家底”搞准弄实。注重增强督查实效，对重大事项、重要工作、重点项目建立清单制度，完善跟踪问效，严格考核奖惩，确保各项工作落实到位。注重提振干事热情，认真落实市委《关于进一步关心关爱和激励干部担当作为干事创业的实施办法（试行）》，给干事者鼓劲，为担当者撑腰，让广大干部愿干事、敢干事、能干成事。

我们要落实全面从严治党责任，切实抓好政府系统党风廉政建设。严格执行中央八项规定及实施细则，驰而不息反对“四风”。全力支持纪委监委工作，坚决反对和抵制一切腐败行为。牢固树立过紧日子的思想，加强财政预算管理，严格控制“三公”经费支出。坚持厉行节约，简化繁文缛节，切实降低行政成本。强化审计监督，加大对项目建设、土地管理、公共资源交易等领域的监管力度，严厉查处发生在群众身边的腐败行为，努力营造风清气正的政治生态。

各位代表，实干成就梦想，奋斗创造幸福。让我们更加紧密地团结在以习近平同志为核心的党中央周围，在习近平新时代中国特色社会主义思想指引下，在自治区党委、政府和市委坚强领导下，锐意进取、砥砺前行，为决胜全面建成小康社会，实现塞上江南、绿色崛起作出新的更大贡献！
